# Nanogenomics and Artificial Intelligence: A Dynamic Duo for the Fight Against Breast Cancer

**DOI:** 10.3389/fmolb.2021.651588

**Published:** 2021-04-15

**Authors:** Batla S. Al-Sowayan, Alaa T. Al-Shareeda

**Affiliations:** Stem Cells and Regenerative Medicine Unit, Cell Therapy & Cancer Research Department, King Abdullah International Medical Research Center, King Saud Bin Abdulaziz University for Health Sciences, Ministry of National Guard Health Affairs, Riyadh, Saudi Arabia

**Keywords:** artificial intelligence, machine learning, exosome, cancer, screening, diagnosis

## Abstract

Application software is utilized to aid in the diagnosis of breast cancer. Yet, recent advances in artificial intelligence (AI) are addressing challenges related to the detection, classification, and monitoring of different types of tumors. AI can apply deep learning algorithms to perform automated analysis on mammographic or histologic examinations. Large volume of data generated by digitalized mammogram or whole-slide images can be interoperated through advanced machine learning. This enables fast evaluation of every tissue patch on an image, resulting in a quicker more sensitivity, and more reproducible diagnoses compared to human performance. On the other hand, cancer cell-exosomes which are extracellular vesicles released by cancer cells into the blood circulation, are being explored as cancer biomarker. Recent studies on cancer-exosome-content revealed that the encapsulated miRNA and other biomolecules are indicative of tumor sub-type, possible metastasis and prognosis. Thus, theoretically, through nanogenomicas, a profile of each breast tumor sub-type, estrogen receptor status, and potential metastasis site can be constructed. Then, a laboratory instrument, fitted with an AI program, can be used to diagnose suspected patients by matching their sera miRNA and biomolecules composition with the available template profiles. In this paper, we discuss the advantages of establishing a nanogenomics-AI-based breast cancer diagnostic approach, compared to the gold standard radiology or histology based approaches that are currently being adapted to AI. Also, we discuss the advantages of building the diagnostic and prognostic biomolecular profiles for breast cancers based on the exosome encapsulated content, rather than the free circulating miRNA and other biomolecules.

For years now, a number of application software are being utilized to aid in the screening and diagnosis of breast cancer. However, these software come with limitations related to the detection, classification, treatment, and monitoring of different types of breast tumors. Therefore, recent advancements in computer science and artificial intelligence (AI) are focusing on addressing these limitations. Unlike previous detection and diagnostic computer-aided software, AI allows the computer to employ algorithms to reach machine-based conclusions in a manner similar to the reasoning-based cognition of the human brain. Machine learning (ML) is a subdomain of AI; ML-based algorithms allow the computer to draw new interfaces based on the available training data (Tran et al., 2019). A branch of ML that is being employed in medical diagnosis is deep learning (DL). DL employs what are called “artificial neural networks” (ANNs). These networks have the structure of a biological nervous system, in that they are multilayer systems where different layers form synaptic connections with each other. Each layer of the network collects input from the lower layer and forms a more complex output. The more the number of layers, the more complex the final output will be (McBee et al., 2018).

For breast cancer diagnosis, DL models are mainly applied to perform analysis on the “gold standard” mammographic examinations. Digitalized mammogram images are interoperated through advanced ML. Most of the DL models used for the analysis are based on the convolutional neural network (CNN) algorithm, which is a class of ANN that is designed to work with data in the form of two-dimensional images (Geras et al., 2019). Several research groups have developed and validated different algorithms using images of breast cancer positive, false-positive, and negative mammogram examinations. Then, they compared the diagnostic performance of certified radiologists with and without the aid of AI. In all of these retrospective studies, the performance of the radiologists, including sensitivity and recall rate and time, was improved when supported by AI (Rodríguez-Ruiz et al., 2019a; Watanabe et al., 2019; Pacil et al., 2020). Until now, there is no single algorithm that could surpass the performance of human radiologists (Schaffter et al., 2020). Nevertheless, scientists continue to improve on previous work, and recent publications are reporting very promising outcomes for AI-based detection as a stand-alone mammography reading practice (Rodríguez-Ruiz et al., 2019b; Shen et al., 2019; Dembrower et al., 2020; Kim et al., 2020; Sasaki et al., 2020). DL models are also being developed for digitalized hematoxylin-eosin (H&E)-stained whole-slide images. This approach is crucial for the detection of malignancies in the breast and other body tissue biopsies including the prostate and the colon. It was reported that AI-based-histological evaluation enabled the fast evaluation of every single tissue patch on the slides of the patients, which led to a performance level comparable to that of a pathologist (Argov et al., 2002; Liu et al., 2019; Shamai et al., 2019; Raciti et al., 2020; Ström et al., 2020). Other groups are attempting to create DL models that rely on non-image data sets. For example, one group used spectral data that reflect changes in collagen, lipids, and nucleic acid content of malignant breast tissues (Dulay et al., 2019). Meanwhile, another group analyzed tissue microarray data in conjunction with DL. This allowed for algorithm-automated gene selection and tumor classification with better performance than when compared to the standard methods (Dashtban and Balafar, 2017).

In addition to the conventional breast cancer screening and diagnostic tests, DL models are also being developed for novel cancer detection. Cancer cell-exomes, which are extracellular vesicles released by cancer cells into the bloodstream, are arising as vital cancer diagnostic and prognostic biomarkers (Al-Sowayan et al., 2019). Exosomes isolated from blood samples of patients with breast cancer were revealed to have characteristic content, mainly proteins and miRNA, which are indicative of the tumor sub-type, possible metastasis, and prognosis (Lee et al., 2019; Tutanov et al., 2020; Wu et al., 2020). However, the utilization of exosomal genetic and proteomic data as a diagnostic tool is confined by the difficulties related to vast data analysis of the multiple molecular biomarkers. Therefore, it is only logical that DL models be developed also for the automated analysis of exosome-related data. One study developed an algorithm for clustering and candidate motif detection in exosomal miRNAs. This study used miRNA sequences downloaded from the “miRBase” database as training and testing data. The results revealed that the algorithm successfully completed the desired function with no human intervention (Gaur and Chaturvedi, 2019). In another study, a DL model that combines the measurement of eight exosomal miRNA biomarkers was used. Then, the model was applied on real-time PCR data of miRNA extracted from exosomes in the plasma of the patients. The algorithm successfully created predictive panels and classified patients with pancreatic cancer from healthy controls (Ko et al., 2017). Meanwhile, in another study, an algorithm that combines the measurement of four exosomal surface biomarkers to detect pancreatic cancer and breast cancer from plasma samples was used. The surface biomarkers were detected with quantitative super-resolution imaging. Then, the model was used to analyze the output of these multiple markers, which led to an accuracy level of 100% (Chen et al., 2019). In addition to exosomal miRNA and surface markers, researchers have also implemented ML in exosomal spectral data, where algorithms were used to build distinctive spectral profiles for cancer exosomes derived from the plasma of patients with lung cancer (Shin et al., 2020) and the saliva of patients with oral cancer (Zlotogorski-Hurvitz et al., 2019); the algorithms yielded an accuracy level of more than 90%.

This nano-AI-based approach could prove to be an advantageous screening and diagnostic method compared to the radiology- or histology-AI-based approaches. Exosomes hold enormous potential in cancer diagnostics as they contain a wealth of proteomic and genetic information. This not only presents a new insight into cancer biology but also enables an elevated level of personalized medicine and thus superior treatment outcomes. Moreover, examining the exosome-encapsulated content as biomarkers is reported to be more reliable compared to examining free-circulating miRNA and other biomolecules. This is due to the fact that exosomal content is highly sensitive to the status of the releasing cancer cell and its microenvironment. In addition, exosome-encapsulated miRNAs are highly stable when compared with free-circulating miRNAs since they are well-protected by the membrane bilayer of the exosome (Yuan et al., 2019). Moreover, the process of blood sample collection is less painful, less dangerous, less time-consuming, less expensive, and does not require a high level of proficiency to obtain when compared to the standard mammograms or biopsies. Theoretically, as proven in principle by the above mentioned studies, exosomes can be isolated from blood samples of patients with breast cancer with known diagnoses and prognoses. Then, the isolated exosome content can be investigated using arrays and analyzed by the computer-aided applications of bioinformatics. When sufficient data are collected, an “exosome-content-signature” for each tumor subtype, the estrogen-receptor statuses, and the possible metastases can be constructed into distinct data sets. These data will serve as the training data for the algorithm to create the DL model. The blood samples are collected from females at any convenient location such as hospitals, family clinics, pharmacies, homes, prisons, and mobile clinics in rural areas. Then, the collected samples are brought back to the designated laboratory with the instrument fit with nano-AI. The instrument will isolate the exomes from the plasma/serum and then extract the data to allow the algorithms to analyze the exosomal composition and match it with the available profiles (Figure 1).

**Figure 1:**
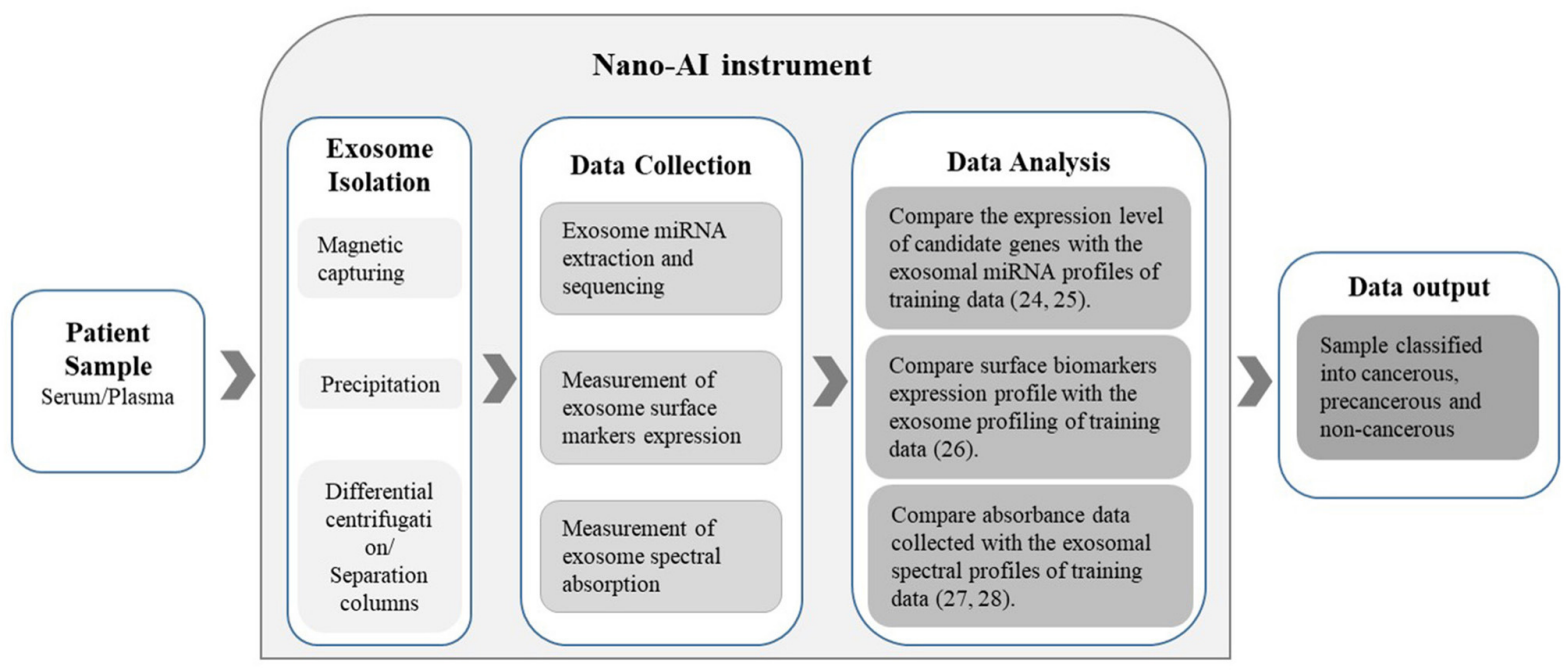
The Nano-AI instrument; schematic illustration of the possible nano-AI instrument workflow for the diagnosis of cancer. Proposed principles of operation include nanogenomics-AI applications and are based on published literature.

Of course, to realize such applications, there are few obstacles that need to be overcome. One main obstacle is the quality control issues related to the isolation and purification of the exosomes from patient samples. Obtaining a consistently pure and measurable yield of exosomes from the sera/plasma of patients is still a work in progress. However, recent advancements in nanotechnology are expected to circumvent all technical issues. Another major obstacle is algorithm creation; it is no doubt that the vast intratumor and interpatient heterogeneities will make it very challenging to assemble distinct molecular profiles for each diagnostic and prognostic variant. Moreover, a major concern in AI applications in general is the algorithm bias that results from using biased training data sets. In medical applications, biased data sets are a consequence of the underrepresentation of certain segments of the population in the evidence base e.g., ethnic minorities (Carter et al., 2020). Therefore, it is crucial to make sure that the clinically adopted algorithm is designed to perform at the highest level with all the patients. This can only be done by using an all-inclusive training data set to create the model, then further validating it using patients with diverse demographic characteristics. In addition to the challenges of exosome isolation and training data creation, there are also general concerns about the utilization of AI in medical applications. These include the ethical and legal matters related to data acquisition such as consenting, confidentiality, ownership, etc. Also, the absence of infrastructure required to adopt the new technology and the fear of increased unemployment among medical professionals are also notable challenges. Nevertheless, the increasing amount of capital invested in the medical applications of AI (investments in start-ups implementing AI in healthcare reached $4 billion in 2019, compared to $2.7 billion in 2018) (Pifer, 2020) is expected to expedite the overcoming of these challenges and encourage a rapid regulation of the ethical, legal, operational, and social issues related to implementation. The clinical value of AI is projected to be fully realized in the very near future. When such nanogenomics-AI-based breast cancer screening approaches are applicable, a wider-spread, early detection of breast cancer will be achieved. This will positively impact the life of every female regardless of her social, economic, or geographical limitations.

## Author Contributions

BA-S conceptualize the idea. All authors contributed to writing and editing the manuscript.

## Conflict of Interest

The authors declare that the research was conducted in the absence of any commercial or financial relationships that could be construed as a potential conflict of interest.

## References

[B1] Al-Sowayan B. S. Al-Shareeda A. T. Al-Hujaily E. M. (2019). Exosomes, cancer's little army. Stem Cell Investig. 6:9. 10.21037/sci.2019.03.02 31119147 PMC6509435

[B2] Argov S. Ramesh J. Salman A. Sinelnikov I. Goldstein J. Guterman H.. (2002). Diagnostic potential of Fourier-transform infrared microspectroscopy and advanced computational methods in colon cancer patients. J. Biomed. Opt. 7, 248–254. 10.1117/1.1463051 11966311

[B3] Carter S. M. Rogers W. Win K. T. Frazer H. Richards B. Houssami N. (2020). The ethical, legal and social implications of using artificial intelligence systems in breast cancer care. Breast 49, 25–32. 10.1016/j.breast.2019.10.001 31677530 PMC7375671

[B4] Chen C. Zong S. Liu Y. Wang Z. Zhang Y. Chen B.. (2019). Profiling of exosomal biomarkers for accurate cancer identification: combining DNA-PAINT with machine-learning-based classification. Small 15:1901014. 10.1002/smll.201901014 31478613

[B5] Dashtban M. Balafar M. (2017). Gene selection for microarray cancer classification using a new evolutionary method employing artificial intelligence concepts. Genomics 109, 91–107. 10.1016/j.ygeno.2017.01.004 28159597

[B6] Dembrower K. Wåhlin E. Liu Y. Salim M. Smith K. Lindholm P.. (2020). Effect of artificial intelligence-based triaging of breast cancer screening mammograms on cancer detection and radiologist workload: a retrospective simulation study. Lancet Digit. Health. 2, e468–e474. 10.1016/S2589-7500(20)30185-0 33328114

[B7] Dulay M. T. Zaman N. Jaramillo D. Mody A. C. Zare R. N. (2019). Rehman, Advancing cancer diagnostics with artificial intelligence and spectroscopy: identifying chemical changes associated with breast cancer. Expert Rev. Mol. Diagn. 19, 929–940. 10.1080/14737159.2019.1659727 31461624

[B8] Gaur P. Chaturvedi A. (2019). Clustering and candidate motif detection in exosomal miRNAs by application of machine learning algorithms. Interdiscip. Sci. Computat. Life Sci. 11, 206–214. 10.1007/s12539-017-0253-4 28733902

[B9] Geras K. J. Mann R. M. Moy L. (2019). Artificial intelligence for mammography and digital breast tomosynthesis: current concepts and future perspectives. Radiology 293, 246–259. 10.1148/radiol.2019182627 31549948 PMC6822772

[B10] Kim H. E. Kim H. H. Han B. K. Kim K. H. Han K. Nam H.. (2020). Changes in cancer detection and false-positive recall in mammography using artificial intelligence: a retrospective, multireader study. Lancet Digit. Health. 2, e138–e148. 10.1016/S2589-7500(20)30003-0 33334578

[B11] Ko J. Bhagwat N. Yee S. S. Ortiz N. Sahmoud A. Black T.. (2017). Combining machine learning and nanofluidic technology to diagnose pancreatic cancer using exosomes. ACS Nano 11, 11182–11193. 10.1021/acsnano.7b05503 29019651

[B12] Lee J. U. Kim W. Y. Lee H. S. Park K. W. Sim S. J. (2019). Quantitative and specific detection of exosomal miRNAs for accurate diagnosis of breast cancer using a surface-enhanced raman scattering sensor based on plasmonic head-flocked gold nanopillars. Small 15:1804968. 10.1002/smll.201804968 30828996

[B13] Liu Y. Kohlberger T. Norouzi M. Dahl G. E. Smith J. L. Mohtashamian A.. (2019). Artificial intelligence–based breast cancer nodal metastasis detection: insights into the black box for pathologists. Arch. Pathol. Lab. Med. 143, 859–868. 10.5858/arpa.2018-0147-OA 30295070

[B14] McBee M. P. Awan O. A. Colucci A. T. Ghobadi C. W. Kadom N. Kansagra A. P.. (2018). Deep learning in radiology. Acad. Radiol. 25, 1472–1480. 10.1016/j.acra.2018.02.018 29606338

[B15] Pacil,è S. Lopez J. Chone P. Bertinotti T. Grouin J. M. Fillard P. (2020). Improving breast cancer detection accuracy of mammography with the concurrent use of an artificial intelligence tool. Radiology 2:e190208. 10.1148/ryai.2020190208 PMC8082372 33937844

[B16] Pifer R. (2020). Health AI Startups Netted a Record $4B in Funding Last Year. Healthcare Dive.

[B17] Raciti P. Sue J. Ceballos R. Godrich R. Kunz J. D. Kapur S.. (2020). Novel artificial intelligence system increases the detection of prostate cancer in whole slide images of core needle biopsies. Modern Pathology 33, 2058–2066. 32393768 10.1038/s41379-020-0551-y PMC9235852

[B18] Rodríguez-Ruiz A. Krupinski E. Mordang J. J. Schilling K. Heywang-Köbrunner S. H. Sechopoulos I.. (2019a). Detection of breast cancer with mammography: effect of an artificial intelligence support system. Radiology 290, 305–314. 10.1148/radiol.2018181371 30457482

[B19] Rodríguez-Ruiz A. Lång K. Gubern-Merida A. Broeders M. Gennaro G. Clauser P.. (2019b). Stand-alone artificial intelligence for breast cancer detection in mammography: comparison with 101 radiologists. JNCI J. Natl Cancer Inst. 111, 916–922. 10.1093/jnci/djy222 30834436 PMC6748773

[B20] Sasaki M. Tozaki M. Rodríguez-Ruiz A. Yotsumoto D. Ichiki Y. Terawaki A.. (2020). Artificial intelligence for breast cancer detection in mammography: experience of use of the ScreenPoint Medical Transpara system in 310 Japanese women. Breast Cancer 27, 642–651. 10.1007/s12282-020-01061-8 32052311

[B21] Schaffter T. Buist D. S. M. Lee C. I. Nikulin Y. Ribli D. Guan Y.. (2020). Evaluation of combined artificial intelligence and radiologist assessment to interpret screening mammograms. JAMA Netw. Open 3:e200265. 10.1001/jamanetworkopen.2020.0265 32119094 PMC7052735

[B22] Shamai G. Binenbaum Y. Slossberg R. Duek I. Gil Z. Kimmel R. (2019). Artificial intelligence algorithms to assess hormonal status from tissue microarrays in patients with breast cancer. JAMA Netw. Open 2:e197700. 10.1001/jamanetworkopen.2019.7700 31348505 PMC6661721

[B23] Shen L. Margolies L. R. Rothstein J. H. Fluder E. McBride R. Sieh W. (2019). Deep learning to improve breast cancer detection on screening mammography. Sci. Rep. 9, 1–12. 10.1038/s41598-019-48995-4 31467326 PMC6715802

[B24] Shin H. Oh S. Hong S. Kang M. Kang D. Ji Y. G.. (2020). Early-stage lung cancer diagnosis by deep learning-based spectroscopic analysis of circulating exosomes. ACS Nano 14, 5435–5444. 10.1021/acsnano.9b09119 32286793

[B25] Ström P. Kartasalo K. Olsson H. Solorzano L. Delahunt B. Berney D. M.. (2020). Artificial intelligence for diagnosis and grading of prostate cancer in biopsies: a population-based, diagnostic study. Lancet Oncol. 21, 222–232. 10.1016/S1470-2045(19)30738-7 31926806

[B26] Tran W. T. Jerzak K. Lu F. I. Klein J. Tabbarah S. Lagree A.. (2019). Personalized breast cancer treatments using artificial intelligence in radiomics and pathomics. J. Med. Imaging Radiat. Sci. 50, S32–S41. 10.1016/j.jmir.2019.07.010 31447230

[B27] Tutanov O. Proskura K. Kamyshinsky R. Shtam T. Tsentalovich Y. Tamkovich S. (2020). Proteomic profiling of plasma and total blood exosomes in breast cancer: a potential role in tumor progression, diagnosis, and prognosis. Front. Oncol. 10:2173. 10.3389/fonc.2020.580891

[B28] Watanabe A. T. Lim V. Vu H. X. Chim R. Weise E. Liu J.. (2019). Improved cancer detection using artificial intelligence: a retrospective evaluation of missed cancers on mammography. J. Digit. Imaging 32, 625–637. 10.1007/s10278-019-00192-5 31011956 PMC6646649

[B29] Wu H. Wang Q. Zhong H. Li L. Zhang Q. Huang Q.. (2020). Differentially expressed microRNAs in exosomes of patients with breast cancer revealed by next-generation sequencing. Oncol. Rep. 43, 240–250. 10.3892/or.2019.7401 31746410 PMC6908931

[B30] Yuan T. B. Li J. Qin J. (2019). Clinical significance of exosomal long noncoding RNA DANCR as a novel serum-based diagnostic and prognostic biomarker in osteosarcoma. Inter J Clin Exp Med. 12, 423–432. Available online at: http://cel.webofknowledge.com/InboundService.do?customersID=atyponcel&smartRedirect=yes&mode=FullRecord&IsProductCode=Yes&product=CEL&Init=Yes&Func=Frame&action=retrieve&SrcApp=literatum&SrcAuth=atyponcel&SID=F2AnypeL7iV7W4Mvk1U&UT=WOS%3A000457202700042

[B31] Zlotogorski-Hurvitz A. Dekel B. Z. Malonek D. Yahalom R. Vered M. (2019). FTIR-based spectrum of salivary exosomes coupled with computational-aided discriminating analysis in the diagnosis of oral cancer. J. Cancer Res. Clin. Oncol. 145, 685–694. 10.1007/s00432-018-02827-6 30603907 PMC11810221

